# Meteorological Register

**Published:** 1834-07-01

**Authors:** 


### METEOROLOGICAL REGISTER,

FROM MARCH 1 TO MAY 31.

By Messrs. HARRIS and Co., Mathematical Instrument Makers, 50, High Holborn.

| | Thermometer. | | Barometer. | | De Luc's Hygrometer. | | Winds. | | Atmospheric Variations. |
|---|---|---|---|---|---|---|---|---|---|
| 1834. | *max.* | *min.* | *max.* | *min.* | *max.* | *min.* | | | |
| Mar. 1 to 7 | 57 | 43 | 30.20 | 29.56 | 80 | 74 | WSW | W | fine, rain, cloudy |
| 14 | 58 | 37 | .26 | 30.07 | 77 | 73 | WNW | NE | fine, foggy, rain |
| 21 | 51 | 32 | .30 | .10 | 71 | 66 | ESE | SE | fine, foggy, cloudy |
| 31 | 55 | 34 | 29.94 | 29.35 | 76 | 66 | WSW | SW | fine, cloudy, rain |
| April 1 to 7 | 59 | 42 | 30.22 | 30.01 | 75 | 65 | NW | NNE | rain, fine, cloudy |
| 14 | 52 | 33 | .12 | 29.76 | 75 | 63 | N | ENE | fine, showery, fine |
| 21 | 62 | 38 | .11 | .93 | 70 | 61 | NNE | ESE | fine, fine, fine |
| 30 | 64 | 40 | .03 | .06 | 74 | 62 | SSW | NNE | fine, rain, cloudy |
| May 1 to 7 | 72 | 49 | 30.15 | .43 | 75 | 64 | SW | SSE | fine, rain, fine |
| 14 | 72 | 52 | .02 | .35 | 69 | 61 | SW | NW | fine, cloudy, rain |
| 21 | 70 | 49 | .20 | .23 | 67 | 61 | SW | NE | fine, showery, cloudy |
| 31 | 72 | 45 | .16 | .81 | 63 | 58 | NE | NNE | fine, fine, cloudy. |

The quantity of Rain fallen in March, 94-100ths }

— — — April, 49-100ths } of an inch.

— — — May, 92-100ths }

